# DNA-delivered monoclonal antibodies targeting the p53 R175H mutant epitope inhibit tumor development in mice

**DOI:** 10.1016/j.gendis.2023.04.027

**Published:** 2023-06-23

**Authors:** Dafei Chai, Xu Wang, Praveen Neeli, Shan Zhou, Xingfang Yu, Kanaga Sabapathy, Yong Li

**Affiliations:** a Department of Medicine, Section of Epidemiology and Population Sciences, Dan L Duncan Comprehensive Cancer Center, Baylor College of Medicine, Houston, TX 77030, USA; b Division of Cellular & Molecular Research, Humphrey Oei Institute of Cancer Research, National Cancer Centre Singapore, Singapore 168583, Singapore

**Keywords:** BsAb, Cytotoxicity, mAb, Mutant p53, PD-1, R175H

## Abstract

The tumor suppressor *p53* is the most common mutated gene in cancer, with the R175H as the most frequent p53 missense mutant. However, there are currently no approved targeted therapies or immunotherapies against mutant p53. Here, we characterized and investigated a monoclonal antibody (mAb) that recognizes the mutant p53-R175H for its affinity, specificity, and activity against tumor cells *in vitro*. We then delivered DNA plasmids expressing the anti-R175H mAb or a bispecific antibody (BsAb) into mice to evaluate their therapeutic effects. Our results showed that the anti-R175H mAb specifically bound to the p53-R175H antigen with a high affinity and recognized the human mutant p53-R175H antigen expressed on HEK293T or MC38 cells, with no cross-reactivity with wild-type p53. In cultured cells, the anti-R175H mAb showed higher cytotoxicity than the control but did not induce antibody-dependent cellular cytotoxicity. We made a recombinant MC38 mouse cell line (MC38-p53-R175H) that overexpressed the human p53-R175H after knocking out the endogenous mutant *p53* alleles. *In vivo*, administration of the anti-R175H mAb plasmid elicited a robust anti-tumor effect against MC38-p53-R175H in mice. The administration of the anti-R175H BsAb plasmid showed no therapeutic effects, yet potent anti-tumor activity was observed in combination with the anti-PD-1 antibody. These results indicate that targeting specific mutant epitopes using DNA-delivered mAbs or BsAbs presents a form of improved natural immunity derived from tumor-infiltrating B cells and plasma cells against intracellular tumor antigens.

## Introduction

Wild-type (WT) p53 is a tumor suppressor that inhibits tumor development via multiple pathways.^1^^,^^2^ Mutations in the *p53* gene occur in approximately 50% of human cancers.^3^^,^^4^ Mutant p53 (mutp53) results in the loss of WT p53-dependent tumor suppressive functions and often the acquisition of oncogenic gain-of-function (GOF) to promote tumor progression and evasion of tumor cell death.^5^^,^^6^ Therapeutic strategies that have been developed to target mutp53, including small compounds, CRISPR/Cas9, small peptides, and immunotherapies, aim to eliminate mutp53 expression or restore the function of WT p53 in tumor cells.^7^, ^8^, ^9^ Although progress has been made, these therapeutic effects are unsatisfactory in the clinic. Currently, there are no effective drugs for mutp53 to address unmet clinical needs.

p53-R175H is a hotspot mutation located in the DNA-binding domain of p53.^10^ This mutation leads to the loss of DNA binding, resulting in resistance to apoptosis, failure of G1 arrest, decrease in genomic stability, and promotion of tumorigenesis.^11^^,^^12^ In addition, the R175H mutant also endows WT p53 with additional functional gains, leading to abnormal activation of gene transcription and enhanced cell migration.^13^ Conversely, suppressing p53-R175H with short hairpin RNA inhibits cell growth, migration, and invasion and weakens the EGFR/PI3K/AKT pathway.^12^^,^^14^ Therefore, the development of drugs specifically targeting p53-R175H presents a potential approach for cancer treatment.

Targeting hotspot mutp53 with an antibody is a promising approach for achieving therapeutic goals. In the past few decades, attempts have been made to generate antibodies against mutp53, and monoclonal antibodies (mAbs) against the conformation of mutp53 (PAb240) or WT p53 (PAb246) have been developed.^15^ However, these antibodies are not specific to mutp53 and exhibit cross-reactivity with WT p53, which limits their therapeutic potential.^16^ In this study, we used a mAb against the R175H of human mutp53 with a high level of specificity and no cross-reactivity to WT p53.^17^ We demonstrated that R175H mAb has potential therapeutic effects *in vivo*. We then designed a T cell-targeting bispecific antibody (BsAb) with dual specificity to the p53-R175H antigen and the mouse CD3 complex. Administration of pR175H-mCD3-BsAb inhibited tumor growth when combined with anti-PD-1 antibody (αPD-1) treatment. These results indicate that anti-mutp53 mAb is an effective treatment for cancers with mutp53.

## Materials and methods

### Cell lines and reagents

Human embryonic kidney (HEK293T) cells, mouse colon cancer cell lines (MC38 and CT26), and a human non-small cell lung cancer cell line (H1299) were obtained from the American Type Culture Collection. HEK293T, MC38, and CT26 cells were cultured in Dulbecco's Modified Eagle Medium (DMEM, Gibco, Grand Island, NY, USA) supplemented with 10% fetal bovine serum (FBS, Gibco) and 1 × anti–anti solution (Gibco). H1299 cells were cultured in Roswell Park Memorial Institute 1640 medium (RPMI-1640, Gibco) supplemented with 10% FBS and 1 × anti–anti solution. The Expi293 cell line was purchased from Thermo Fisher Scientific and cultured in Freestyle™ 293 Expression Medium (Gibco) at 125 rpm with 8% CO_2_ at 37 °C. MC38-p53-R175H cells (stably overexpressing human p53-R175H after endogenous p53 knockout) and CT26-p53-R172H cells (p53-R172H knockin) were constructed using lentivirus and cultured in complete DMEM. The antibodies used in this study are as follows: Anti-p53 antibody (clone DO-1, Santa Cruz Biotechnology, Santa Cruz, CA, USA), anti-β-actin antibody (clone AC-74, Sigma–Aldrich, Saint Louis, MO, USA), anti-p53 antibody (clone 1C12, Cell Signaling Technology, Danvers, MA, USA), and anti-PD-1 antibody (αPD-1) (clone RMP1-14, BioXcell, West Lebanon, NH, USA), anti-mouse CD16/32 antibody (clone 93, BioLegend, San Diego, CA, USA), Brilliant Violet 750™-conjugated anti-mouse Cd45 (clone 30-F11, BioLegend), and PE-conjugated anti-human IgG Fc (clone M1310G05, BioLegend).

### Constructs expressing the antibodies

The R175H antibody plasmids encoding the heavy-chain and light-chain^15^ were synthesized, and the antibody was purified by Syd Labs, Inc. The single-chain variable fragment (scFv) was obtained from a previous study.^17^ Assembly of antibody heavy- and light-chain DNAs into a mammalian expression vector pTwist (Twist Bioscience, South San Francisco, CA, USA) or gWIZ (Aldevron, Fargo, ND, USA) was performed to construct pR175H-mAb or pR175H/mCD3-BsAb. The plasmids were transformed into competent *Escherichia coli* cells and propagated in Luria–Bertani (LB) broth supplemented with 100 μg/mL ampicillin (Teknova, Inc., Hollister, CA, USA) or 50 μg/mL kanamycin (Teknova, Inc.). Plasmids were purified from DH5α cells grown overnight using an endotoxin-free ZymoPURE™ II Plasmid Maxiprep Kit (Zymo Research, Irvine, CA, USA).

### Antibody expression and purification

The antibodies were expressed using the ExpiFectamine™ 293 Transfection Kit (Gibco) following the manufacturer's instructions. Briefly, Expi293 cells were cultured in 30 mL of Expi293 expression medium on an orbital shaker (125 rpm) at 37 °C in 8% CO_2_. Expi293 cells were prepared at 3 × 10^6^/mL and then diluted with 30 μg of plasmid encoding pR175H-mAb or pR175H-BsAb in 1.5 mL of Opti-MEM (Gibco), and 90 μL of ExpiFectamine™ 293 reagent (1.5 mL). The two diluents were mixed, incubated at room temperature for 20 min, and added to the cells. Transfection enhancers were added 24 h post-transfection, and the cells were cultured for five days. Afterward, cells were removed by centrifugation at 800 *g* for 5 min, and the supernatant of the culture medium was harvested and concentrated at 4 °C using 10 kDa MWCO (Cytiva, Marlborough, MA, USA).

The antibodies were purified using the NAb Protein G Spin Column kit (Thermo Scientific, Waltham, MA, USA) following the manufacturer's protocol. The columns were balanced with 2 mL of binding buffer. A volume of 200 μL concentrated medium supernatant was diluted to 2 mL in binding buffer and incubated with end-over-end mixing for 10 min. The columns were washed three times with 2 mL binding buffer. Finally, 1 mL elution buffer was used for elution. Elution was performed three times, and fractions were collected in three 15 mL tubes containing 100 μL neutralization buffer. The purified samples were dialyzed against PBS in Slide-A-Lyzer dialysis cassettes (Thermo Fisher Scientific) at 4 °C overnight. The samples were analyzed using SDS-PAGE and quantified using the Pierce BCA Protein Assay Kit (Thermo Fisher Scientific).

### Biolayer interferometry (BLI)

The binding of His-tagged TrxA-R175H peptides to R175H mAbs or R175H/mCD3-BsAbs was detected using BLI (Gator Bio, Palo Alto, CA, USA) with a Ni-NTA-biosensor probe. Antigens and antibodies were exchanged into Q Buffer (PBS at pH 7.4, 0.02% Tween-20, 0.2% BSA, and 0.05% NaN3). TrxA-R175H-peptide (Analyte) and TrxA-R282W peptide (negative control) were diluted in Q buffer, while R175H-mAb or R175H-BsAb was diluted in Q buffer and loaded onto the Ni-NTA sensor chip. Initially, the Ni-NTA Biosensor was hydrated in 200 μL Q buffer for 10 min and then exposed to 250 μL Q buffer to obtain an initial baseline reading. The Ni-NTA biosensor was then dipped into the R175H-mAb or R175H-BsAb for 120 s (loading). After loading, the biosensor was exposed to 200 μL Trx-R175H or TrxA-R282W-peptide for 30 s to obtain another baseline measurement. The biosensor was then exposed for 120 s to obtain an association curve. This resulted in a binding–association curve. Finally, the biosensor was exposed to 250 μL Q buffer to obtain dissociation measurements. After each cycle, the sensor was regenerated using a regeneration buffer, Gly-HCl (pH 1.5). The data were reference subtracted and fitted to a 1:1 binding model (Rmax global fit) using Gator Data Analysis Software (Gator Bio).

### Western blot

Samples were lysed using RIPA buffer with a protease and phosphatase inhibitor cocktail (Thermo Fisher Scientific) and pelleted at 12,000 rpm for 15 min at 4 °C. The supernatants were collected, and concentration was quantified using the BCA Protein Assay Reagent (Thermo Fisher Scientific). The samples were separated using Tris-glycine SDS-PAGE (4%–20% polyacrylamide, Mini-PROTEAN Precast Gels, Bio-Rad, Hercules, CA, USA), and then transferred onto a polyvinyl difluoride membrane. The membrane was blocked with 5% non-fat milk for 2 h at room temperature and then incubated with the primary antibody at 4 °C overnight, followed by the secondary anti-IgG horseradish peroxidase-linked antibody (Cell Signaling Technology). The bands were developed using a western blotting substrate (Thermo Fisher Scientific).

### Cytotoxicity assay

Peripheral blood mononuclear cells (PBMCs) were purified from human buffy coats (Gulf Coast Regional Blood Center, Houston, TX, USA) using Ficoll-Hypaque (GE Healthcare, Chicago, IL, USA). To determine the cytotoxicity, 1 × 10^4^ cells (MC38 or H1299) expressing human p53-R175H were co-cultured with or without PBMCs in the presence of anti-p53-R175H mAb at different indicated concentrations in 96-well plates (Corning, Corning, NY, USA) at 37 °C for 72 h. Cytotoxicity was determined by measuring the amount of lactate dehydrogenase in the supernatant using the Cytotoxicity Detection Kit PLUS (Roche Applied Science, Indianapolis, IN, USA). Cytotoxicity was calculated as (experimental value − low control)/(high control − low control) × 100%.

### ELISA

ELISA plates were coated with p53-R175H antigen, which was dissolved in coated buffer (R&D Systems, Minneapolis, MN, USA) overnight at 4 °C. The plates were washed three times with PBST (pH 7.4) containing 0.05% (v/v) Tween-20 and then blocked with 3% BSA in PBS for 1 h. Serum was then added and incubated at room temperature for 2 h. The binding was detected using a horseradish peroxidase-conjugated second antibody (Cell Signaling Technology). The reaction was developed using a TMB substrate (R&D Systems) and then stopped with 2 N H_2_SO_4_. The absorbance at 450–650 nm was measured using a plate reader (CLARIOstar, BMG Labtech, USA).

### *In vivo* studies

C57BL/6J and BALB/c female mice (six weeks old) were purchased from Jackson Laboratory (Bar Harbor, ME, USA) and maintained in the animal facility of Baylor College of Medicine under pathogen-free conditions. All procedures were performed with the approval of the Institutional Animal Care and Use Committee (IACUC) of Baylor College of Medicine. A syngeneic mouse colon cancer model was prepared by inoculating MC38-p53-R175H or CT26-p53-R172H cells subcutaneously. The pR175H-mAb, pR175H-BsAb, or control plasmid was administered intramuscularly by electroporation using TriGrid Delivery System (TDS-IM) device (Ichor Medical Systems, San Diego, CA, USA) at a specified time point. Meanwhile, combination therapy with αPD-1 was administered after electroporation. The tumor volumes were measured and calculated as (length × width^2^)/2.

### Flow cytometry assessments

The tumor tissues were excised and minced into approximately 1 mm^3^ cubic pieces. They were then digested using a mouse tumor dissociation kit (Miltenyi Biotec) and incubated on a rocker (Gentle MACS Octo 8, Miltenyi Biotec) at 37 °C for 25–40 min. The resulting digested cells were filtered through 70-μm cell strainers (BD Pharmingen), subjected to red blood cell lysis, and washed twice with cold PBS containing 2% FBS. After blocking Fc receptors and removing dead cells with a Zombie Aqua Fixable Viability Kit (BioLegend), cells were stained with anti-mouse Cd45 and anti-human IgG Fc for 30 min at room temperature and in the dark. Finally, the samples were analyzed using a Cytek NL-3000 flow cytometry system, and the data were analyzed using FlowJo V10 (BD Biosciences).

### Statistical analyses

Data are expressed as mean ± standard deviation (SD) unless otherwise stated. Two groups were compared using a two-tailed independent Student's *t* test. GraphPad Prism 8.0 (GraphPad Software, San Diego, CA, USA) was used for statistical analysis. *P* values < 0.05 were considered statistically significant (^∗^*P* < 0.05, ∗∗*P* < 0.01, ∗∗∗*P* < 0.001, ∗∗∗∗*P* < 0.0001).

## Results

### Characteristics and specificity of anti-p53-R175H mAb

**Figure 1:**
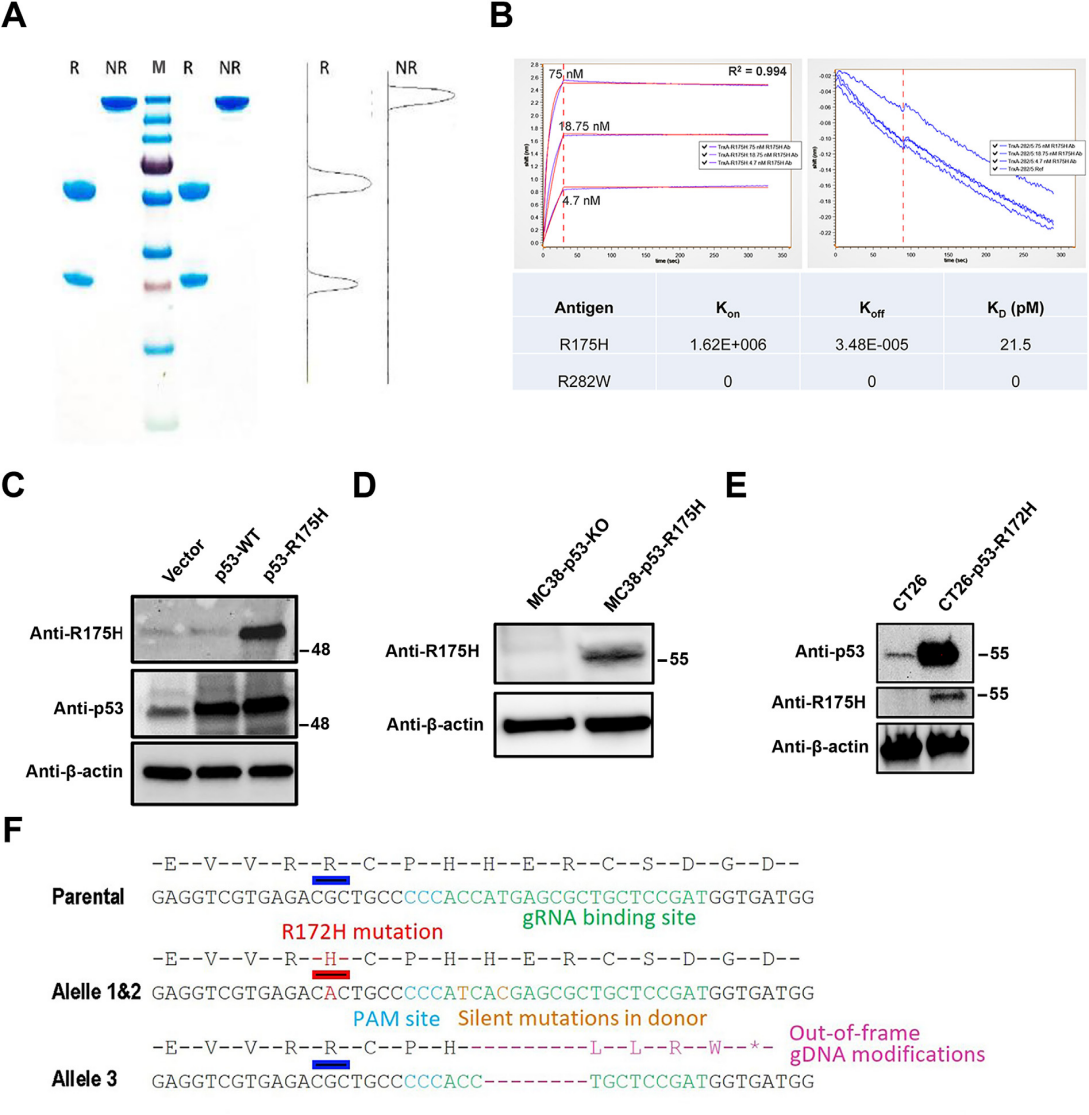
Characterization of p53-R175H mAb. **(A)** The purified anti-R175H protein was separated on a 4%–20% SDS-PAGE gel and stained with Coomassie solution. Lanes 1 and 4 were loaded with reducing anti-R175H mAb; Lanes 2 and 5 were loaded with non-reducing protein; Lane 3 indicates the protein ladder (250, 130, 100, 70, 55, 35, 25, 15, 10 kDa, Thermo Fisher Scientific). **(B)** BLI kinetics of R175H mAb association (*t* = 0 s–120 s) and dissociation (*t* > 120 s) with R175H antigen. **(C)** Forty-8 h after transfection of 293T cells with WT p53, p53-R175H, or the vector control, a Western blot was used to evaluate the expression of WT p53 or p53-R175H. **(D)** The protein expression levels of p53-R175H were detected using Western blot in MC38 cells without p53 or with human p53-R175H. **(E)** The mutp53 protein expression in CT26-p53-R172H cells was detected by Western blot with the R175H mAb. **(F)** Knocking the R172H mutation into mouse *p53* gene in CT26 cells.

The anti-R175H mAb was purified from culture supernatant of 293Expi cells that were transiently transfected with two plasmids expressing the heavy chain and the light chain of the mAb. The protein content was assessed using reducing and non-reducing SDS-PAGE analyses. In the presence of β-mercaptoethanol, the heterodimer anti-R175H band was separated into a heavy chain and a light chain, which migrated at approximately 50 kDa and 25 kDa, respectively (lanes 1 and 4; Fig. 1A). Moreover, the purity of the antibody was > 96% (Fig. 1A). As shown in Figure 1B, the binding kinetics of anti-R175H mAb to the R175H antigen was determined using BLI at room temperature. The anti-R175H mAb showed an affinity to the R175H antigen^17^ with a dissociation constant of 21.5 pM.

Western blot was performed to further confirm whether the anti-R175H mAb bound specifically to p53-R175H. The anti-R175H mAb exhibited a strong specific recognition with the R175H protein in p53-R175H 293T cells but did not bind to the WT p53 protein or protein extracts from p53 knockout MC38 cells (Fig. 1C, D), as previously reported.^17^ Moreover, the anti-R175H mAb also recognized mouse p53-R172H protein in CT26-p53-R172H cells (Fig. 1E). The CT26-p53-R172H cell line was modified from CT26, which has 3 WT *p53* alleles.^18^ We designed the gRNA and a repair donor with R172H (CGC > CAC) point mutation and two adjacent silent mutations (H175H, CAC > CAT; H176H, CAT > CAC) to prevent gRNA-mediated cleavage of the repair donor or the correctly edited genome (Fig. 1F). The resulting clones were sequenced and validated with IB using R175H-specific mAbs. We obtained a clone with p53^R172H/R172H/−^ and named the line CT26-p53-R172H. These results indicate that anti-R175H mAb binds explicitly to human p53-R175H or mouse p53-R172H but has no cross-reactivity with WT p53. This is expected, as the adjacent 18 amino acids around the mutated R residue are identical in human and mouse p53.^17^

### Anti-p53-R175H mAb showed limited cytotoxicity against R175H-positive tumor cells *in vitro*

**Figure 2:**
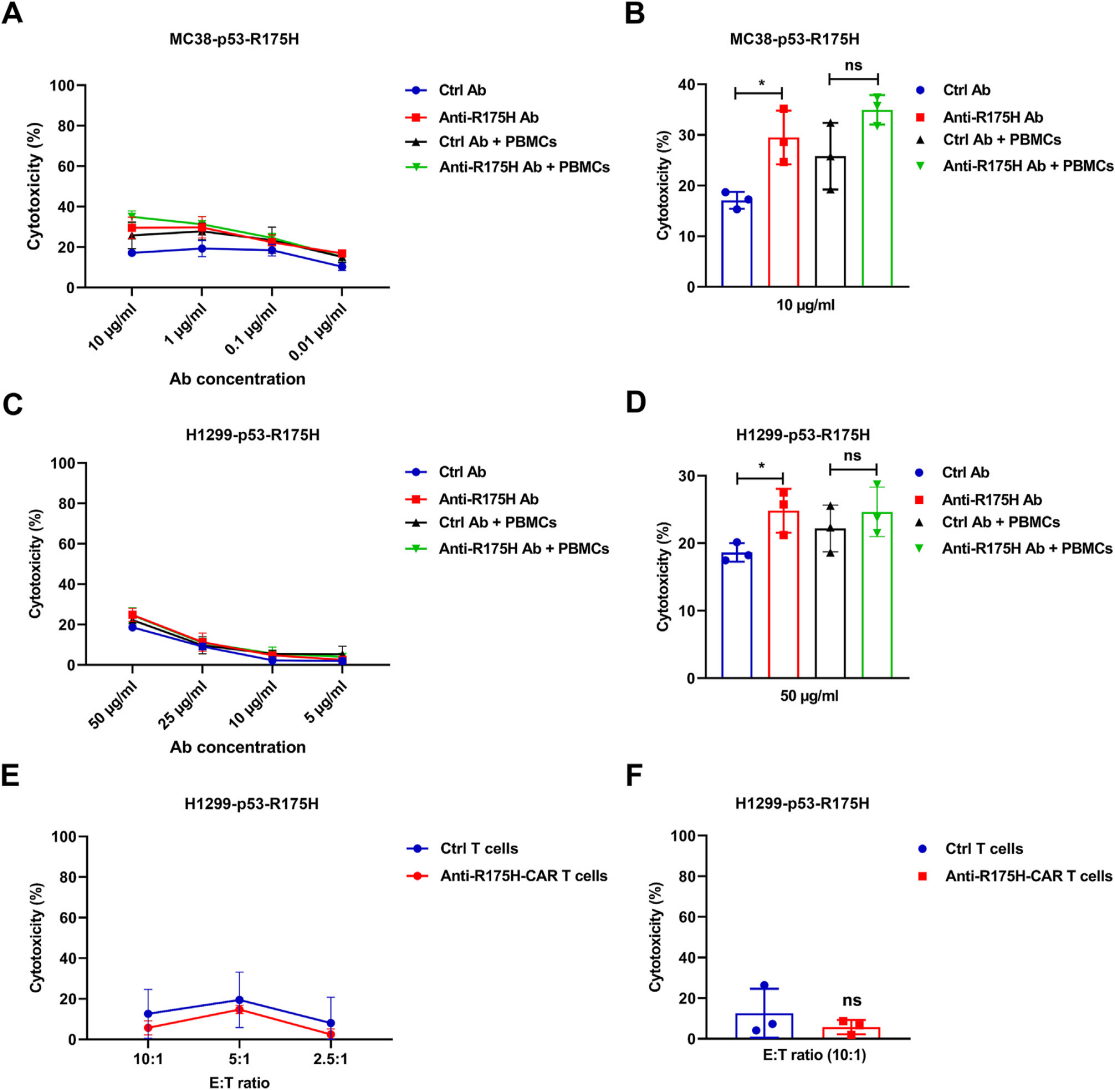
The cytotoxicity of R175H mAb in cultured cells. MC38-p53-R175H or H1299-p53-R175H cells were added into a 96-well tissue culture plate at a density of 1 × 10^4^ cells/well in the presence of various concentrations of R175H mAb or isotype control. PBMCs (1 × 10^5^ cells/well) were co-cultured with tumor cells in a humidified incubator with 5% CO_2_ at 37 °C for three days. **(A)** At the mAb concentration of 10, 1, 0.1, and 0.01 μg/mL, the cytotoxicity of MC38-p53-R175H cell supernatant was measured using the lactate dehydrogenase (LDH) assay. **(B)** Cytotoxicity was determined using the LDH assay with 10 μg/mL mAb. **(C, D)** The cytotoxicity of the H1299-R175H cell supernatant was measured using the LDH assay after cells were incubated with 10, 1, 0.1, and 0.01 μg/mL mAb. **(E, F)** H1299-p53-R175H cells (1 × 10^4^ cells) harboring a luciferase reporter gene were co-cultured with CAR-T cells at various concentrations in 96-well plates at 37 °C overnight. Cytotoxicity was determined by measuring the amount of luciferase in lysed target cells. The data shown are representative of three experiments. The data are expressed as mean ± SD. Statistical significance was set at ^∗^*P* < 0.05. ns, not significant.

To investigate whether the anti-p53-R175H mAb suppresses tumor cell proliferation, we co-cultured MC38-p53-R175H cells and PBMCs in the presence or absence of the mAb. The MC38-p53-R175H cells were generated by knocking out the endogenous p53 mutant alleles (G242V & S238I) and introducing the human p53 gene with the R175H mutation. We noted moderate cytotoxicity of the anti-p53-R175H mAb at the highest concentration (10 μg/mL) towards MC38-p53-R175H cells compared to the isotype control-treated cells; however, we did not observe statistically significant differences in the cytotoxicity of anti-p53-R175H mAb-treated cells in the presence of PBMCs (Fig. 2A, B). Limited cytotoxicity was also observed in the anti-R175H mAb-treated H1299 overexpressing the human p53-R175H mutant (Fig. 2C, D). No significant difference was observed between the anti-p53-R175H mAb and the combination of the mAb and PBMCs, indicating that the mAb did not induce cytotoxicity by antibody-dependent cellular cytotoxicity. Next, we cloned the scFv of the anti-p53-R175H mAb into a chimeric antigen receptor (CAR) construct and evaluated the cytotoxicity of mutp53-R175H CAR T cells against H1299 cells overexpressing p53-R175H. The mutp53-R175H CAR T cells showed no cytotoxicity compared to the control CAR T cells (Fig. 2E, F). Collectively, these results indicate that the anti-p53-R175H mAb showed limited cytotoxicity against mutp53-R175H tumor cells in the antibody-dependent cellular cytotoxicity and CAR T-cell settings.

### The anti-p53-R175H mAb encoded by DNA or combined with αPD-1 mAb treatment inhibited tumor growth *in vivo*

**Figure 3:**
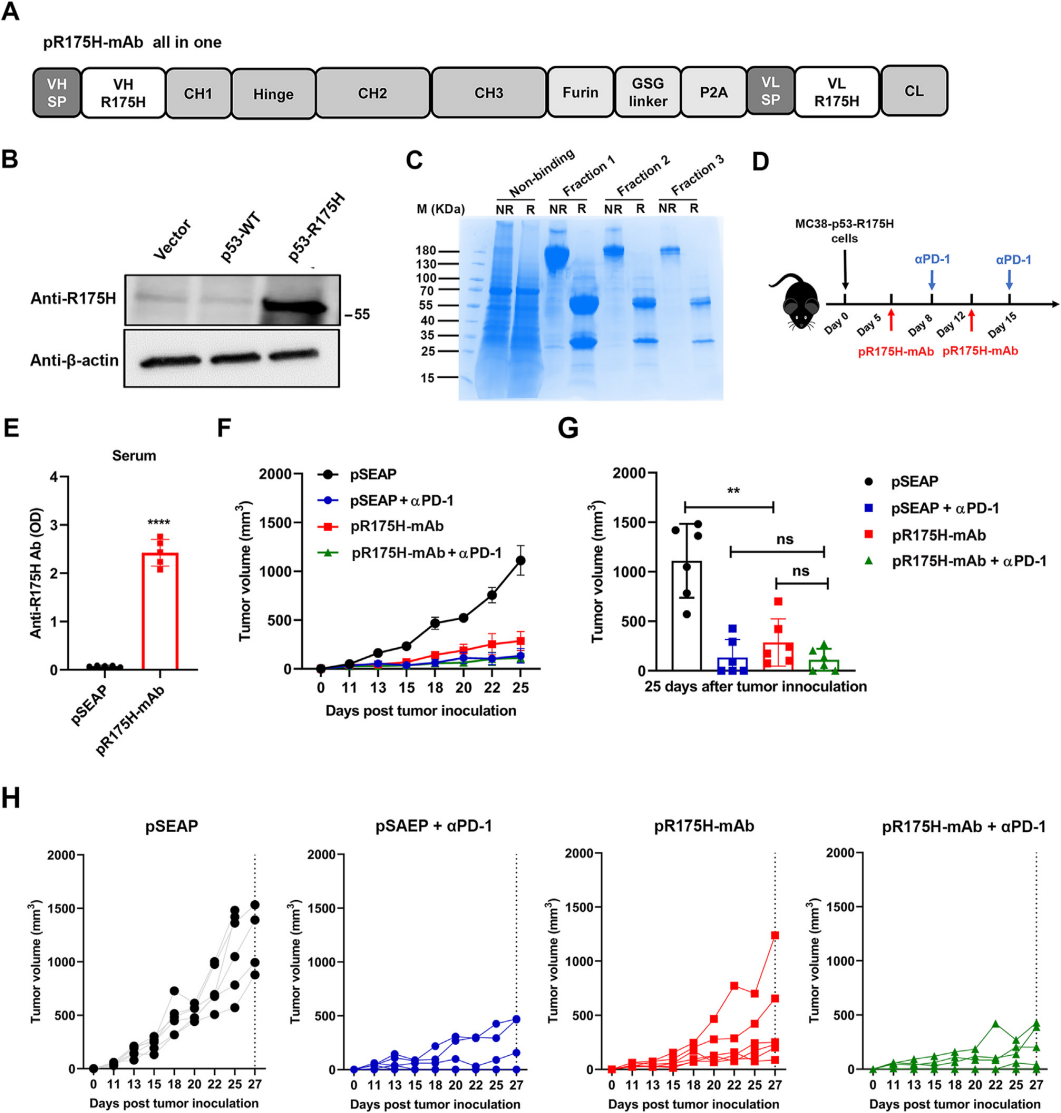
The therapeutic effect of pR175H-mAb in mouse MC38 cell tumor model. **(A)** Schema of the pR175H-mAb all-in-one construct. **(B)** The expression of R175H was detected using a Western blot with anti-p53-R175H mAb in 293T cells transfected with WT p53 or p53-R175H. **(C)** Purified protein from pR175H-mAb-transfected 293 cells was separated on an SDS-PAGE gel and stained with Coomassie solution. **(D)** Schema of MC38-R175H tumor model establishment and experiment. The subcutaneous MC38-R175H cell tumor model was intramuscularly inoculated with pR175H-mAb, followed by electroporation on days 5 and 12 after tumor inoculation. Meanwhile, αPD-1 was administrated intraperitoneally on days 8 and 15. **(E)** The serum levels of anti-R175H Ab were detected using ELISA. **(F)** Tumor volume was measured at different times after inoculation. The data are expressed as mean ± SEM. **(G)** Tumor volume was statistically analyzed on day 25 post-tumor inoculation. **(H)** Tumor diameter of individual mice from the groups in (F) as a function of time. Six mice in each group were tested. The data shown are representative of three experiments. The data are expressed as mean ± SD. ^∗∗^*P* < 0.01 and ^∗∗∗∗^*P* < 0.0001. ns, not significant.

We next constructed a single plasmid (pR175H-mAb) to express the full mAb (Fig. 3A). Western blot showed that the anti-p53-R175H mAb exhibited a strong specific recognition with p53-R175H protein but not WT p53 protein expressed in 293T cells (Fig. 3B). The anti-p53-R175H mAb purified from the pR175H-mAb-transfected 293Expi cell system was evaluated by reducing and non-reducing SDS-PAGE analyses (Fig. 3C). We used the MC38-p53-R175H tumor model to evaluate the anti-tumor properties of the pR175H-mAb and αPD-1 in a therapeutic setting (Fig. 3D). Mice were injected subcutaneously with MC38-p53-R175H cells, and 200 μg of pR175H-mAb DNA per animal was injected intramuscularly before electroporation on days 5 and 12 post-tumor inoculations. αPD-1 (200 μg and 100 μg) per animal was administered on days 8 and 15. As shown in Figure 3E, the pR175H-mAb group showed a significant increase in anti-R175H Ab on day 5 after a single electroporation injection compared to the control groups with the parental vector expressing SEAP (pSEAP). Treatment with two doses of pR175H-mAb post-tumor challenge significantly reduced tumor growth compared to the control (Fig. 3F–H). αPD-1 alone also significantly reduced tumor volumes. However, we did not observe significant enhancement with the combination of pR175H-mAb and αPD-1.

**Figure 4:**
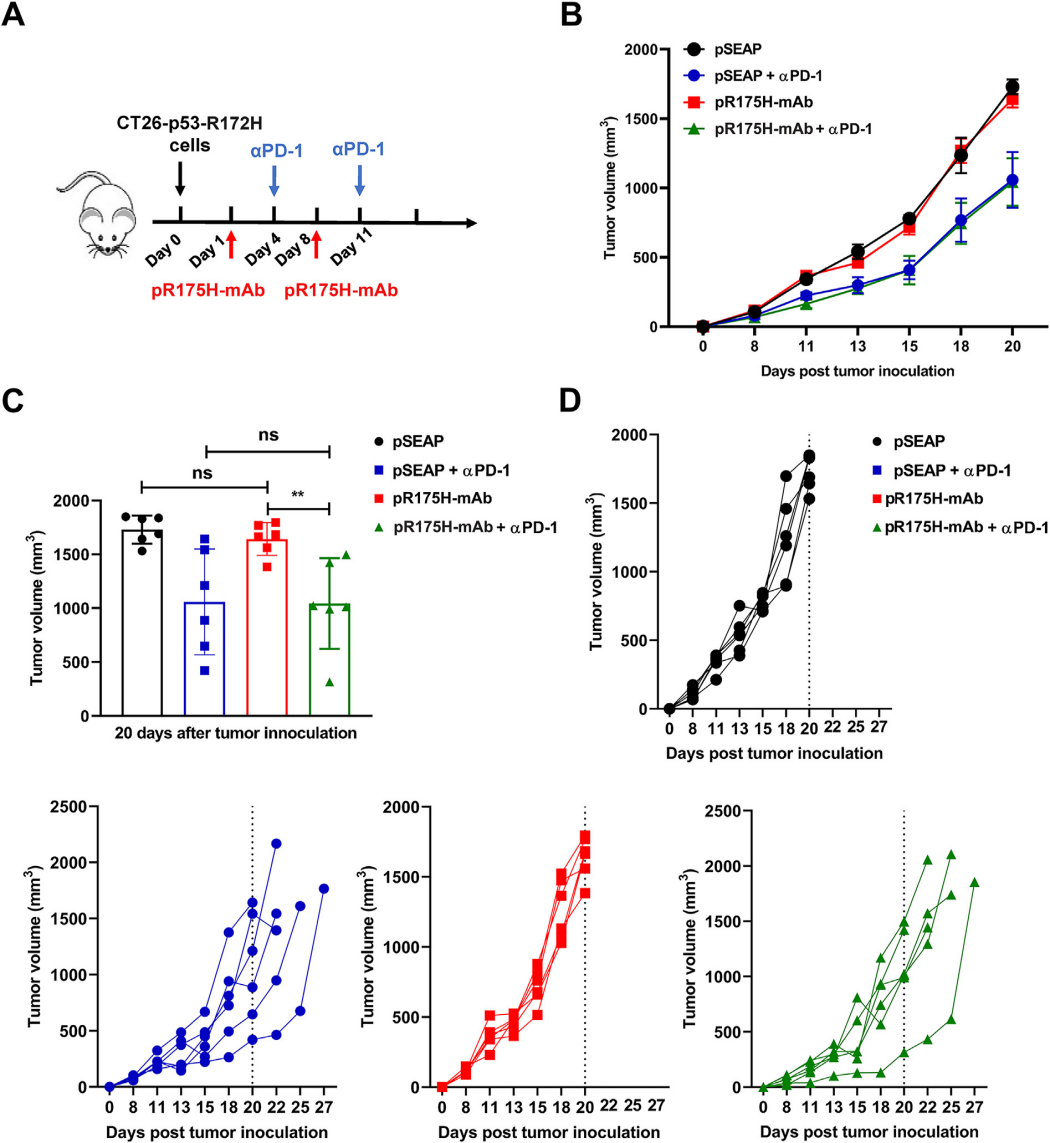
Anti-tumor response induced by pR175H-mAb on CT26 tumor model. **(A)** Schema of CT26-R172H tumor model establishment and experiment. The subcutaneous tumor model was established and intramuscularly inoculated with pR175H-mAb on days 1 and 8 after tumor inoculation. Meanwhile, αPD-1 was administrated intraperitoneally on days 4 and 11. Each group included six mice. **(B)** Tumor growth was measured and compared on the indicated days after initial inoculation. The data were expressed as mean ± SEM. **(C)** Tumor volume was statistically analyzed on day 20 post-tumor inoculation. **(D)** Tumor volume of individual mice from the groups in (B) as a function of time. The data are shown as mean ± SD. ^∗∗^*P* < 0.01. ns, not significant.

To further validate the anti-tumor efficacy of pR175H-mAb, we used another mouse tumor line. The endogenous WT *p53* gene was mutated to p53-R172H, resulting in the CT26-p53-R172H recombinant line. We performed intramuscular injection and electroporation of pR175H-mAb on days 1 and 8 post-tumor inoculation and intraperitoneal administration of αPD-1 (200 μg each) on days 4 and 11 (Fig. 4A). αPD-1 but not the pR175H-mAb inhibited tumor development; the combination treatment did not augment the therapeutic benefit of αPD-1 (Fig. 4B–D). These results suggest that pR175H-mAb alone may have cell line-specific effects on the MC38-p53-R175H cells.

### DNA-based anti-p53-R175H BsAb combined with αPD-1 treatment exerts potential anti-tumor efficacy

**Figure 5:**
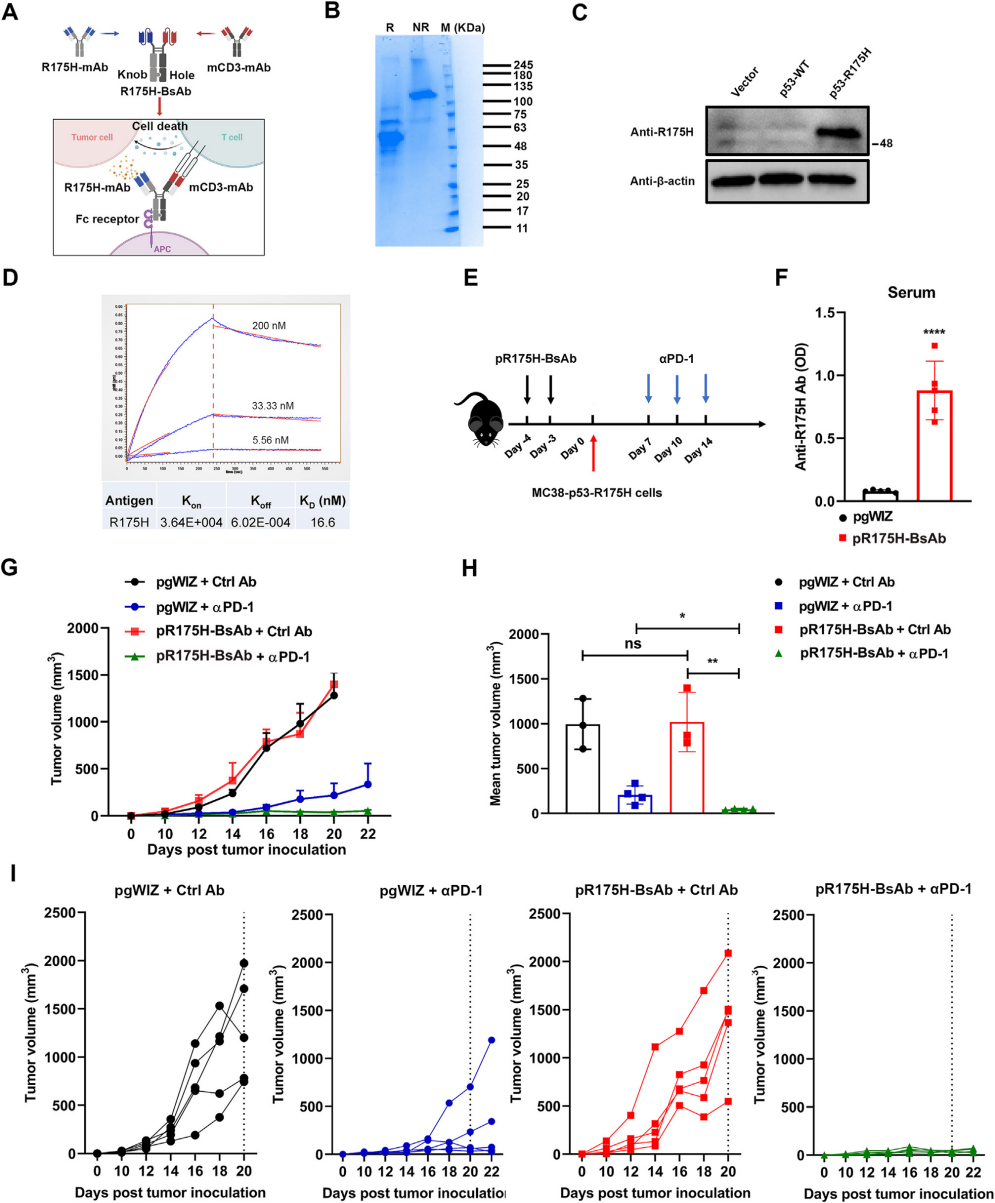
The therapeutic effect of pR175H-BsAb was evaluated *in vivo*. **(A)** Schema of the pR175H-BsAb construct using the knob-into-hole technology. **(B)** The purified protein from pR175H-BsAb transfection in 293 cells was separated on an SDS-PAGE gel and stained with Coomassie solution. **(C)** The expression of p53-WT or R175H was detected using a Western blot with anti-R175H BsAb in 293T cells transfected with an empty vector or that expressing p53-WT or p53-R175H. **(D)** BLI kinetics of R175H/mCD3 mAb association (*t* = 0 s–120 s) and dissociation (*t* > 120 s) with R175H antigen. **(E)** Schema of MC38-R175H tumor model establishment and experiment. MC38-R175H subcutaneous tumor model was intramuscularly inoculated with pR175H-BsAb on days 4 and 3 before tumor inoculation. Meanwhile, three doses of αPD-1 were administrated intraperitoneally on days 7, 10, and 14. **(F)** The serum levels of anti-R175H/mCD3 BsAb were detected using an ELISA. **(G)** Tumor volume was measured at different times after inoculation. The data are expressed as mean ± SEM. **(H)** Statistical analysis of mean tumor volume was performed on days 16–22 post-tumor inoculation. **(I)** Tumor diameter of individual mice from the groups in (G) as a function of time. The experiments were performed with five mice per group. The data are expressed as mean ± SD. ^∗^*P* < 0.05, ^∗∗^*P* < 0.01, and ^∗∗∗∗^*P* < 0.0001.

The pR175H-BsAb was engineered by combining the p53-R175H scFv with an anti-mouse CD3 scFv fused to a human immunoglobulin Fc domain (Fig. 5A). The pR175H-BsAb was purified from the supernatant of transiently transfected Expi293 cells by a standard protein G chromatography. As shown in Figure 5B, the purified BsAb was correctly assembled through reduced and non-reduced SDS-PAGE. Next, the binding specificity of R175H-BsAb was assessed by Western blot, which showed that the BsAb exhibited a strong specific recognition with the mutp53-R175H protein but did not bind with WT p53 protein expressed in 293T cells (Fig. 5C). The R175H-BsAb displayed a decent affinity for the mutp53-R175H antigen, and its dissociation constant was 16.6 nM (Fig. 5D). We then performed intramuscular injection and electroporation of the pR175H-BsAb. Compared to the control group, the pR175H-BsAb-treated mice had a higher level of anti-R175H antibody, demonstrating that pR175H-BsAb was efficiently expressed *in vivo* (Fig. 5F).

We next delivered two doses of pR175H-BsAb on days 4 and 3 to mice that were inoculated with MC38-p53-R175H cells on day 0. Three 200-μg doses of αPD-1 were delivered on days 7, 10, and 14 (Fig. 5E). There was no significant difference in tumor growth in pR175H-BsAb-treated mice compared to the control, implying that pR175H-BsAb alone has no therapeutic benefit. When combined with αPD-1 treatment, the pR175H-BsAb treatment showed a more substantial therapeutic effect than αPD-1 alone in suppressing tumor growth in mice (Fig. 5G–I). Collectively, these results suggest that pR175H-BsAb may enhance the therapeutic benefits of αPD-1 treatment.

**Figure 6:**
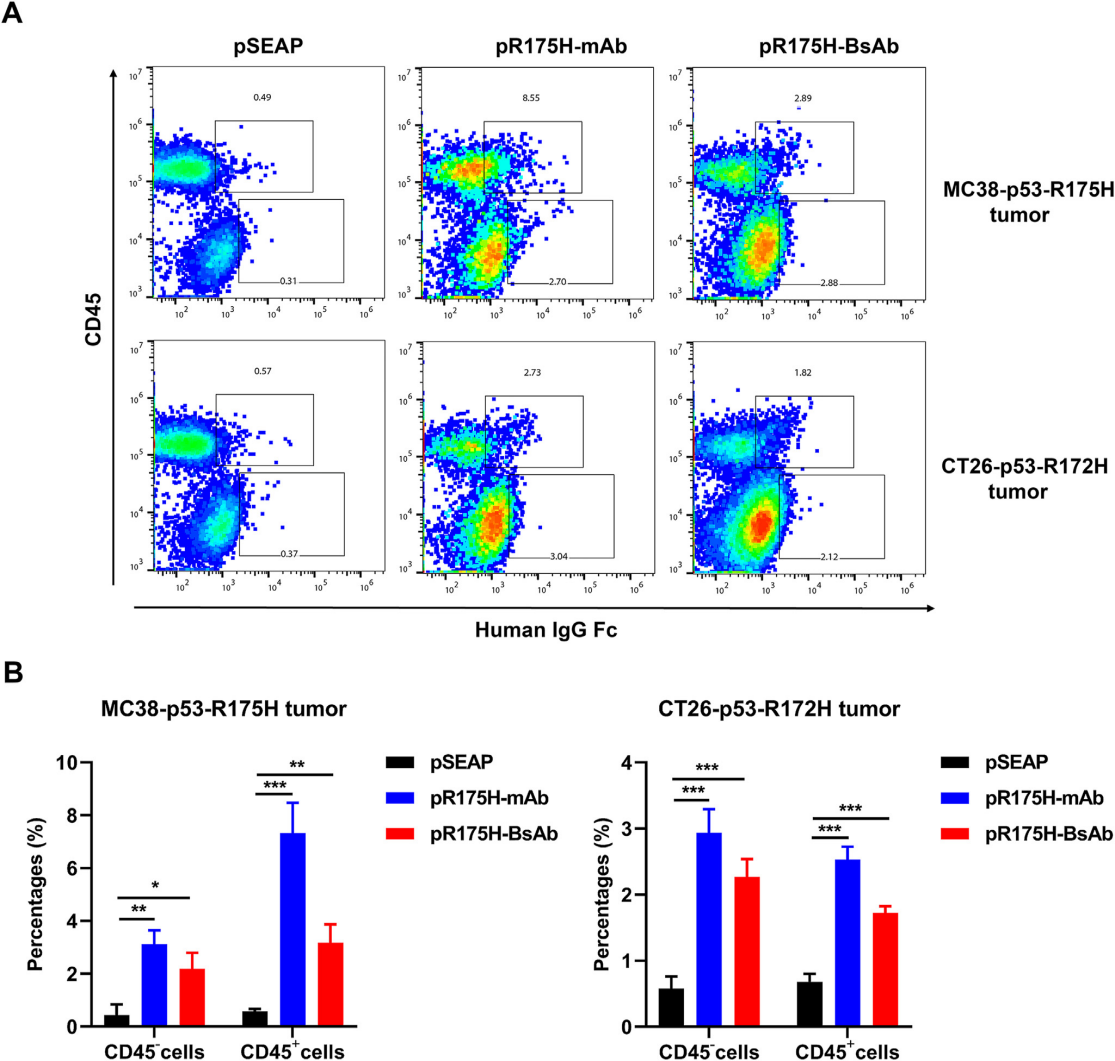
Target antigen recognized by pR175H-mAb or BsAb *in vivo*. The subcutaneous tumors (MC38-p53-R175H on C57BL/6J and CT26-p53-R175H on BALB/c) were established before mice were intramuscularly inoculated with pSEAP, pR175H-mAb, or pR175H-BsAb. Tumor tissues were collected 7 days after the last treatment, and single-cell suspension was subjected to flow cytometry using antibodies against human IgG Fc and mouse Cd45. The experiments were performed with three mice per group. **(A)** The frequency of human Fc-positive Cd45^+^ and Cd45^−^ cells from tumors. Representative images were shown. **(B)** Statistical analysis of cells that were stained positive by the anti-Fc antibody. The data are shown as mean ± SD. ^∗^*P* < 0.05, ^∗∗^*P* < 0.01, ^∗∗∗^*P* < 0.001.

To assess whether DNA-based anti-p53-R175H mAb or BsAb could target the mutant antigen presented on tumor cells *in vivo*, we administered pR175H-mAb, pR175H-BsAb, or the control plasmid intramuscularly by electroporation in MC38-p53-R175H or CT26-p53-R172H models. After three doses of treatments, we used flow cytometry to detect the cells from tumor tissues using antibodies directed against mouse Cd45 and human IgG Fc. In both tumor models, the pR175H-mAb-treated group exhibited increased staining by the anti-Fc antibody for Cd45^−^ cells and Cd45^+^ immune cells compared to the control (Fig. 6). Similarly, pR175H-BsAb-treated tumors also showed increased staining, although the percentage of positive cells was lower. These findings suggest that some mutant p53-R175H epitopes, either short peptides or full-length mutant proteins, were presented on the tumor cell surface. The Fc receptors on Cd45^+^ cells could bind the mAb and BsAb and be stained by the anti-Fc antibody. The fewer neoantigens on the cell surface in CT26 tumors likely contribute to the negative results.

## Discussion

Mutp53-R175H is the most common p53 mutation in cancer,^19^ accounting for 3.69% or 4.21% of all cancers with TP53 mutations, based on data from the TCGA program or the IARC TP53 somatic mutations database. Yet, there is no anti-mutp53 drug approved by the FDA. In this study, we constructed a plasmid expressing both the heavy and light chains of a previously reported anti-R175H-mAb^17^ and evaluated its therapeutic benefits against MC38 tumors overexpressing the human p53-R175H mutant. The DNA-delivered anti-R175H mAb inhibited tumor development of MC38-p53-R175H, which was not enhanced by αPD-1 treatment. We then constructed a cell line, CT26-p53-R172H, in which endogenous WT p53 was mutated to mutp53-R172H. Interestingly, the administration of pR175H-mAb did not reduce tumor growth from CT26-p53-R172H compared to the control. The αPD-1 antibody reduced the tumorigenesis of CT26-R172H, yet the combination of αPD-1 and pR175H-mAb showed no improvement over αPD-1 alone. To enhance pR175H-mAb-mediated T cell anti-tumor immunity, a BsAb was designed to recognize the p53-R175H epitope and mouse CD3. The affinity of the BsAb to the p53 antigen was only 16.6 nM, with an approximately 770-fold reduction from 21.5 pM of the mAb. pR175H-BsAb was used to treat mice inoculated with MC38-p53-R175H, leading to reduced tumorigenesis. Mice treated with pR175H-BsAb combined with αPD-1 showed stronger suppression of tumorigenesis than either agent alone. These results indicate that pR175H-BsAb induced T-cell anti-tumor immunity *in vivo*. A bispecific single-chain antibody, named H2-scDb, binds to the specific mutant p53-R175H peptide attached to an MHC (major histocompatibility complex)-I and recruits T cells.^20^ H2-scDb can activate T cells and consequently eliminate cancer cells with the specific p53-R175H peptide. Our BsAb is MHC-independent and does not need a continuous pump for delivery. The use of human Fc in our mAb and BsAb design for mouse experiments may limit their therapeutic efficacy due to the mice's anti-human immune response.

Treating cancer patients with checkpoint-inhibiting antibodies is clinically successful, but the high cost may limit their use for patients in low-income countries.^21^ The high cost is caused by manufacturing complexity, patents, and large amounts (grams) per patient.^22^ BsAbs that bring two different scFvs together into one molecule present a challenge from an expression and a manufacturing perspective. The knob-into-hole technology has been widely adopted to produce BsAbs. DNA plasmids encoding mAb and BsAb are easy to prepare and are effective in targeting the p53-R175H neoantigen. We use an intramuscular injection of the plasmids before electroporation because of the controlled delivery, accessibility, ease of operation, and fewer systemic effects. The peak serum levels of mAb and BsAb in mice reach ∼30 and 4 μg/mL, comparable to the reported 9.1–19.7 μg/mL after one infusion of the αPD-1 antibody pembrolizumab in humans.^23^ Thus, targeting tumor-specific and tumor-associated antigens by DNA-delivered mAbs and BsAbs could be a viable and affordable therapeutic method for cancer patients and beyond.^24^^,^^25^

Mutp53 elicits both humoral and cellular immune responses in experimental animals and human patients. The discovery of p53 is partially based on the humoral response in mammals to mutp53. Simian vacuolating virus 40 (SV40)-transformed mouse cells stimulate the production of a cellular 53kD protein that is specifically immunoprecipitated with sera from mice, hamsters, or rabbits bearing the SV40-induced tumors.^26^^,^^27^ This protein (*i.e.*, p53) is also found in the sera of mice bearing tumors induced by chemicals, irradiation, or spontaneous tumors.^28^ Thus, p53 has long been recognized as cellular tumor antigen p53^29^, ^30^, ^31^ or antigen NY-CO-13.^32^ Right after the discovery of p53, anti-p53 antibodies are found in the sera of ∼9% of breast cancer patients.^33^ Overall, they are detectable in cancer patients with a specificity of ∼96%, but the sensitivity of anti-p53 serum antibodies to predict p53 mutation is only ∼30%.^34^ In virtually all detections for anti-p53 antibodies in cancer patients' sera, the WT p53 was used as the antigen.^35^ Recently, mounting evidence supports that tumor-infiltrating B cells and plasma cells (collectively called tumor-infiltrating B lymphocytes) have a crucial and multifaceted role in tumor control.^36^ Tumor-infiltrating B lymphocytes promote anti-tumor immunity in most cancers through cell-based and antibody-based effector mechanisms.^36^^,^^37^ There are about 70 antigens recognized by tumor-infiltrating B lymphocyte-derived antibodies,^36^ with p53 as a major antigen.^38^, ^39^, ^40^, ^41^, ^42^ Tumor-infiltrating B lymphocyte-derived antibodies and serum-derived autoantibodies against most antigens may originate and persist independently in cancer patients.^36^^,^^37^ Tumor-infiltrating B lymphocyte-derived antibodies are likely produced locally within tertiary lymphoid structures that arise *de novo* in hot tumors.^36^^,^^42^ CD8^+^ cytotoxic T lymphocytes are the primary effector cells for anti-tumor immune responses. They recognize neoantigens presented on the tumor cell surface by MHC-I molecules, leading to the killing of tumor cells. CD4^+^ T-helper cells orchestrate and sustain the local immune attack by cytotoxic T lymphocytes. In contrast, CD4^+^ FoxP3^+^ regulatory T cells impede anti-tumor immunity by inhibiting cytotoxic T lymphocyte activation. Spontaneous MHC-I-restricted p53-specific cytotoxic T lymphocytes^43^^,^^44^ and MHC-II-restricted p53-specific proliferating T-helper cells^45^, ^46^, ^47^ are found in mice and patients, supporting that proteolysis of the intracellular p53 protein in tumors results in the presentation of p53-derived peptides restricted by MHCs at the tumor cell surface. Recently, T-cell receptors targeting mutp53-derived neoantigens are cloned and proposed as therapeutic agents.^48^, ^49^, ^50^, ^51^ Overall, these data support that cancer patients have both humoral and cellular immune responses against mutp53 in their tumors, underlying the scientific premise to develop personalized p53-targeting agents by improving natural immunity. Leveraging the antibody-mediated effects of tumor-infiltrating B lymphocytes has been proposed for safe and effective therapies by screening and engineering neoantigen-specific mAbs to minimize the autoimmune sequelae.^36^ Such antibodies in the form of IgA in the ovarian cancer microenvironment contribute to thwarting malignant progression.^52^ The engineered anti-p53 mAb and BsAb in the IgG isotype here represent a form of improved natural immunity derived from tumor-infiltrating B lymphocytes against this cellular tumor antigen.

In summary, our results demonstrated the ability of pR175H-mAb or BsAb to target mutant R175H and inhibit tumor development in the MC38 murine syngeneic mouse model. Combination therapy of pR175H-mAb or BsAb with αPD-1 has significant potential for cancer treatment. mAb and BsAb should be considered novel strategies to tackle undruggable oncoproteins with accessible mutant epitopes. Even without therapeutic success, the developed mAbs will offer extraordinary values in cancer companion or complementary diagnostics (*e.g.*, to detect mutant p53 protein levels for therapeutics based on T-cell receptors^48^, ^49^, ^50^, ^51^ or small compound inhibitors^53^, ^54^, ^55^, ^56^). The mAbs and BsAbs against mutant p53 will become increasingly valuable as membrane-disruption- and carrier-based technologies continue to expand the frontiers of intracellular macromolecule delivery.^57^

## Author contributions

DC, XW, and YL conceived and designed the project. XW and DC performed the project and analyzed the data. DC, XW, PN, SZ, XY, KS, and YL contributed reagents, materials, and analysis tools and wrote, reviewed, and edited the manuscript. All authors read and approved the final manuscript.

## Conflict of interests

The authors declare that there is no conflict of interests.

## Funding

YL is a CPRIT Scholar in Cancer Research supported by the Cancer Prevention and Research Institute of Texas (No. RR190043).
